# Supplementary material for: From sequence to enzyme mechanism using multi-label machine learning
Source: BMC Bioinformatics. 2014 May 19;15:150. doi: 10.1186/1471-2105-15-150 (PMC4229970; doi:10.1186/1471-2105-15-150)
Supplement: Additional file 2 — Java code of ml2db. Additional file ml2db_code.tar.gz contains the Java source code to run the multi-label machine learning experiments and save the results to database. The code’s Javadoc is included. [file 1471-2105-15-150-S2.zip › additional file 2/ml2db/utils/doc/index-files/index-18.html]

R-Index


JavaScript is disabled on your browser.


- Overview
- Package
- Class
- Use
- Tree
- Deprecated
- Index
- Help

- Prev Letter
- Next Letter

- Frames
- No Frames

- All Classes

A B C D E F G H I J K L M N O P Q R S T U V W X Y 


## R

RandomApp - Class in edu.cornell.lassp.houle.RngPack
:   RandomApp is a simple application that demonstrates the use of RngPack.

RandomApp() - Constructor for class edu.cornell.lassp.houle.RngPack.RandomApp


RandomElement - Class in edu.cornell.lassp.houle.RngPack
:   RandomElement is an abstract class that encapsulates uniform random number
    generators.

RandomElement() - Constructor for class edu.cornell.lassp.houle.RngPack.RandomElement


RandomEngine - Class in cern.jet.random.engine
:   Abstract base class for uniform pseudo-random number generating engines.

RandomJava - Class in edu.cornell.lassp.houle.RngPack
:   RandomJava is a class wrapper for the `Math.random()` generator
    that comes with Java.

RandomJava() - Constructor for class edu.cornell.lassp.houle.RngPack.RandomJava


RandomSeedable - Class in edu.cornell.lassp.houle.RngPack
:   `RandomSeedable` is an abstract class that extends the
    `RandomElement` class to include the ability to automatically
    generate a valid `long` seed from the clock.

RandomSeedable() - Constructor for class edu.cornell.lassp.houle.RngPack.RandomSeedable


RandomShuffle - Class in edu.cornell.lassp.houle.RngPack
:   RandomShuffle uses one random number generator to shuffle the numbers
    produced by another to obliterate sequential correlations.

RandomShuffle(RandomElement, RandomElement, int) - Constructor for class edu.cornell.lassp.houle.RngPack.RandomShuffle


RandomUtilsTest - Class in uk.ac.standrews.utils.test.stats
:   Class

RandomUtilsTest() - Constructor for class uk.ac.standrews.utils.test.stats.RandomUtilsTest


Ranecu - Class in edu.cornell.lassp.houle.RngPack
:   Ranecu is an advanced multiplicative linear congruential random number
    generator with a period of aproximately 10^18^.

Ranecu() - Constructor for class edu.cornell.lassp.houle.RngPack.Ranecu
:   Initialize RANECU with the default seeds from James.

Ranecu(Date) - Constructor for class edu.cornell.lassp.houle.RngPack.Ranecu


Ranecu(int, int) - Constructor for class edu.cornell.lassp.houle.RngPack.Ranecu
:   Initialize RANECU with two specified integer seeds.

Ranecu(long) - Constructor for class edu.cornell.lassp.houle.RngPack.Ranecu


Ranlux - Class in edu.cornell.lassp.houle.RngPack
:   RANLUX is an advanced pseudo-random number generator based on the
    RCARRY algorithm proposed in 1991 by Marsaglia and Zaman.

Ranlux() - Constructor for class edu.cornell.lassp.houle.RngPack.Ranlux
:   Default initialization of RANLUX.

Ranlux(Date) - Constructor for class edu.cornell.lassp.houle.RngPack.Ranlux
:   Initialize RANLUX with default luxury
    level and a Date object.

Ranlux(int) - Constructor for class edu.cornell.lassp.houle.RngPack.Ranlux
:   Initialize RANLUX with default luxury
    level and a specified seed.

Ranlux(int, Date) - Constructor for class edu.cornell.lassp.houle.RngPack.Ranlux
:   Initialize RANLUX with specified luxury
    level and a Date object.

Ranlux(int, int) - Constructor for class edu.cornell.lassp.houle.RngPack.Ranlux
:   Initialize RANLUX with specified luxury
    level and seed.

Ranlux(int, long) - Constructor for class edu.cornell.lassp.houle.RngPack.Ranlux
:   Initialize RANLUX with specified luxury
    level and seed.

Ranlux(long) - Constructor for class edu.cornell.lassp.houle.RngPack.Ranlux
:   Initialize RANLUX with default luxury
    level and a specified seed.

Ranmar - Class in edu.cornell.lassp.houle.RngPack
:   RANMAR is a lagged Fibonacci generator proposed by Marsaglia and
    Zaman and is a good research grade generator.

Ranmar() - Constructor for class edu.cornell.lassp.houle.RngPack.Ranmar
:   Initialize Ranmar with a default seed taken from Marsaglia and Zaman's
    paper.

Ranmar(Date) - Constructor for class edu.cornell.lassp.houle.RngPack.Ranmar
:   Seed RANMAR from the clock.

Ranmar(int) - Constructor for class edu.cornell.lassp.houle.RngPack.Ranmar
:   Initialize Ranmar with a specified integer seed

Ranmar(long) - Constructor for class edu.cornell.lassp.houle.RngPack.Ranmar
:   Initialize Ranmar with a specified long seed

raw() - Method in class cern.jet.random.engine.RandomEngine
:   Returns a 32 bit uniformly distributed random number in the open unit
    interval `(0.0,1.0)` (excluding 0.0 and 1.0).

raw() - Method in class edu.cornell.lassp.houle.RngPack.RandomElement
:   Returns a uniformly distributed pseudo-random number.

raw(double[]) - Method in class edu.cornell.lassp.houle.RngPack.RandomElement
:   Fill an entire array with doubles.

raw(double[], int) - Method in class edu.cornell.lassp.houle.RngPack.RandomElement
:   Fill part or all of an array with doubles.

raw() - Method in class edu.cornell.lassp.houle.RngPack.RandomJava
:   Wrapper for `Math.random().`

raw() - Method in class edu.cornell.lassp.houle.RngPack.RandomShuffle
:   The generator.

raw() - Method in class edu.cornell.lassp.houle.RngPack.Ranecu


raw(double[], int) - Method in class edu.cornell.lassp.houle.RngPack.Ranecu
:   This is an inline version that returns an array of doubles for speed.

raw() - Method in class edu.cornell.lassp.houle.RngPack.Ranlux
:   Returns a uniformly distributed pseudo-random double in the range (0,1).

raw() - Method in class edu.cornell.lassp.houle.RngPack.Ranmar
:   Returns a uniformly distributed pseudo-random double in the range (0,1).

raw(double[], int) - Method in class edu.cornell.lassp.houle.RngPack.Ranmar
:   A version of the generator for filling arrays, inlined for speed

readConnectionProperties(String) - Method in class uk.ac.standrews.utils.main.database.DbConn
:   Reads the connection properties from the java properties file specified
    in the path

readLocalProperties(String) - Static method in class uk.ac.standrews.utils.main.PropertiesUtils
:   Reads properties that inherit from three locations.

readLocalProperties(String) - Static method in class uk.ac.standrews.utils.main.Utils
:   Reads properties that inherit from three locations.

readProperties(String) - Static method in class uk.ac.standrews.utils.main.PropertiesUtils
:   Reads properties Code adapted from Weka:
    http://www.cs.waikato.ac.nz/ml/weka/
    Ian H.

readProperties(String) - Static method in class uk.ac.standrews.utils.main.Utils
:   Reads properties Code adapted from Weka:
    http://www.cs.waikato.ac.nz/ml/weka/
    Ian H.

redirectStdErr(File) - Static method in class uk.ac.standrews.utils.main.LogUtils
:   Redirect stderr to file

ReflectionUtils - Class in uk.ac.standrews.utils.main
:   Class

ReflectionUtils() - Constructor for class uk.ac.standrews.utils.main.ReflectionUtils


ReflectionUtilsTest - Class in uk.ac.standrews.utils.test
:   Class

ReflectionUtilsTest() - Constructor for class uk.ac.standrews.utils.test.ReflectionUtilsTest


refreshConnection() - Method in class uk.ac.standrews.utils.main.database.DbManager


regenerateConnection() - Method in class uk.ac.standrews.utils.main.database.DbConn


RegExpUtils - Class in uk.ac.standrews.utils.main


RegExpUtils() - Constructor for class uk.ac.standrews.utils.main.RegExpUtils


RegExpUtilsTest - Class in uk.ac.standrews.utils.test
:   Class

RegExpUtilsTest() - Constructor for class uk.ac.standrews.utils.test.RegExpUtilsTest


removeAttribute(String) - Method in class uk.ac.standrews.utils.main.webutils.simpledomparser.XmlNode
:   remove an attribute by name

removeChildNode(XmlNode) - Method in class uk.ac.standrews.utils.main.webutils.simpledomparser.XmlNode
:   Remove a child element

removeEmptyKeys(TreeMap<String, String>) - Static method in class uk.ac.standrews.utils.main.maputils.MapUtils


removeEmptyString(TreeSet<String>) - Static method in class uk.ac.standrews.utils.main.collectionutils.setutils.SetUtils


removeEmptyStrings(Collection<String>) - Static method in class uk.ac.standrews.utils.main.collectionutils.CollectionUtils


removeEmptyStrings() - Method in class uk.ac.standrews.utils.main.maputils.OneToManyMap


removeKey(T) - Method in class uk.ac.standrews.utils.main.maputils.OneToManyMap


removeValue(T, U) - Method in class uk.ac.standrews.utils.main.maputils.OneToManyMap
:   Remove a value from the key's list of values

removeValue(U) - Method in class uk.ac.standrews.utils.main.maputils.OneToManyMap
:   Remove a value from \*all\* the key's list of values

removeValues(Collection<U>) - Method in class uk.ac.standrews.utils.main.maputils.OneToManyMap


ResultSetUtils - Class in uk.ac.standrews.utils.main.database
:   Class

ResultSetUtils() - Constructor for class uk.ac.standrews.utils.main.database.ResultSetUtils


resultsSetToString(ResultSet) - Static method in class uk.ac.standrews.utils.main.database.DbUtils
:   Generates a string for the results of a query

REVERSED\_PRIME\_CHARACTER - Static variable in class uk.ac.standrews.utils.main.database.DbUtils


rightRemoveString(String, String) - Static method in class uk.ac.standrews.utils.main.stringutils.StringUtils


roundDouble(int, Double) - Static method in class uk.ac.standrews.utils.main.NumberUtils
:   From http://stackoverflow.com/questions/153724/how-to-round-a-number-to-n-decimal-places-in-java

roundDouble(double, int) - Static method in class uk.ac.standrews.utils.main.Utils
:   Rounds a number to just n decimal digits

runAnalyzeTable() - Method in class uk.ac.standrews.utils.main.database.DbManager
:   Runs the 'ANALYZE TABLE tablename' SQL command for all the tables in a
    mysql database.

A B C D E F G H I J K L M N O P Q R S T U V W X Y

- Overview
- Package
- Class
- Use
- Tree
- Deprecated
- Index
- Help

- Prev Letter
- Next Letter

- Frames
- No Frames

- All Classes
